# 

**Published:** 1865

**Authors:** 


### NOTICES.

The fifth annual meeting of the AMERICAN DENTAL ASSOCIATION will be held at Chicago, Ill., on Tuesday, July 25th, 1865, at 10 o'clock, A. M.

It seems almost unnecessary to impress upon delegates the importance of their presence, since the very large attendance last session was a signal demonstration of the interest and sympathy of the profession, which of itself will undoubtedly prove sufficient to establish the popularity of an Association based upon representative interest.

The preparation of voluntary essays is at this time urged upon delegates not occupying positions upon any of the various committees, as even such contributions will serve to render the proceedings additionally interesting and profitable.

In connection with the ordinary exercises, clinics will be held and various dental operations will be performed.

GEO. W. ELLIS, *Cor. Sec'y.*

---

The second annual meeting of the CENTRAL STATES ASSOCIATION OF DENTAL SURGEONS will be held at Louisville, Ky., at the Kentucky Medical College, commencing on Tuesday, July 18th, at 10 o'clock, A. M.

W. H. SHADOAN, *Secretary*

---

The next regular meeting of the IOWA STATE DENTAL SOCIETY will take place at Dubuque, Iowa, beginning July 18th. Those attending it can go from it to the NATIONAL ASSOCIATION, which meets July 25th. We cordially invite any of our Eastern brethren to come to our meeting; they can easily do so by stating to the Association one week sooner than they otherwise would. WM. O. KULP, *Cor. Sec.*

*Muscatine, Iowa, June* 1, 1865.

### Editorial.

### AMERICAN DENTAL CONVENTION.

The American Dental Convention meets on the first Tuesday of August next at White Sulphur Springs, Ohio. This place we regard as a very excellent selection. None more central could have been chosen; it is about equal distance between the East and the West. The facility of reaching it from all points is good. It is not directly upon the railroad. Its railroad station is upon the Columbus & Cleveland Railroad, about fifteen miles north of Columbus.

The Springs are some four or five miles from the railroad, but ample and comfortable accommodations are always at hand for the conveyance of passengers. It is a beautiful locality, though nothing either magnificent or grand in the scenery, but it is a fine, pleasant place, and one just adapted for such a meeting, for there are not attractions outside to draw members away; and doubtless the efficiency and value of these meetings depend much upon the attention which the members give during the session. We anticipate a large outpouring of the profession at the approaching convention.

### AMERICAN DENTAL ASSOCIATION.

This body meets at Chicago on the 25th of July prox., when we anticipate a very large meeting of the profession. There are already a large number of members of this Association and there will be a large number additional this year, by the introduction of new delegates. Quite a number of new societies have been organized within the last year; they, we doubt not, will be fully represented, and all the societies heretofore represented have been flourishing and growing, so that it is legitimate to expect a far larger attendance this year than ever before. The influence for good of this Central Association is so manifest, that almost all see and appreciate it. We hope all will go prepared to take part in its proceedings. And we would especially suggest that all societies prepare and present their reports, according to the resolution of last year; we hope it will not be omitted by any, as such reports would be very interesting. A new feature —clinic exercises—is to be introduced, which will doubtless prove highly interesting and instructive. There will be other demonstrations and illustrations, which will be advantageous to all. Those who witnessed them will remember the demonstrations of last year with pleasure and profit.

